# Supplementary material for: Predictive model for cytoneme guidance in Hedgehog signaling based on Ihog- Glypicans interaction
Source: Nat Commun. 2022 Sep 26;13:5647. doi: 10.1038/s41467-022-33262-4 (PMC9512826; doi:10.1038/s41467-022-33262-4)
Supplement: Supplementary file 1 — Supplementary Information [file 41467_2022_33262_MOESM1_ESM.pdf]

Supplementary information

Supplementary figures:

- **Supplementary figure 1:** Cytoneme stabilization and orientation in abdominal histoblast nets.
- **Supplementary figure 2:** Orientation of posterior compartment cytonemes under Ihog levels.
- **Supplementary figure 3:** Cytoneme stabilization depends on Ihog levels in confronted cell populations.
- **Supplementary figure 4:** Molecular competition between the available Ihog and glypicans for cytoneme stabilization and orientation.

Supplementary note:

- Mathematical-physical model for the evolution of continuous cytonemes

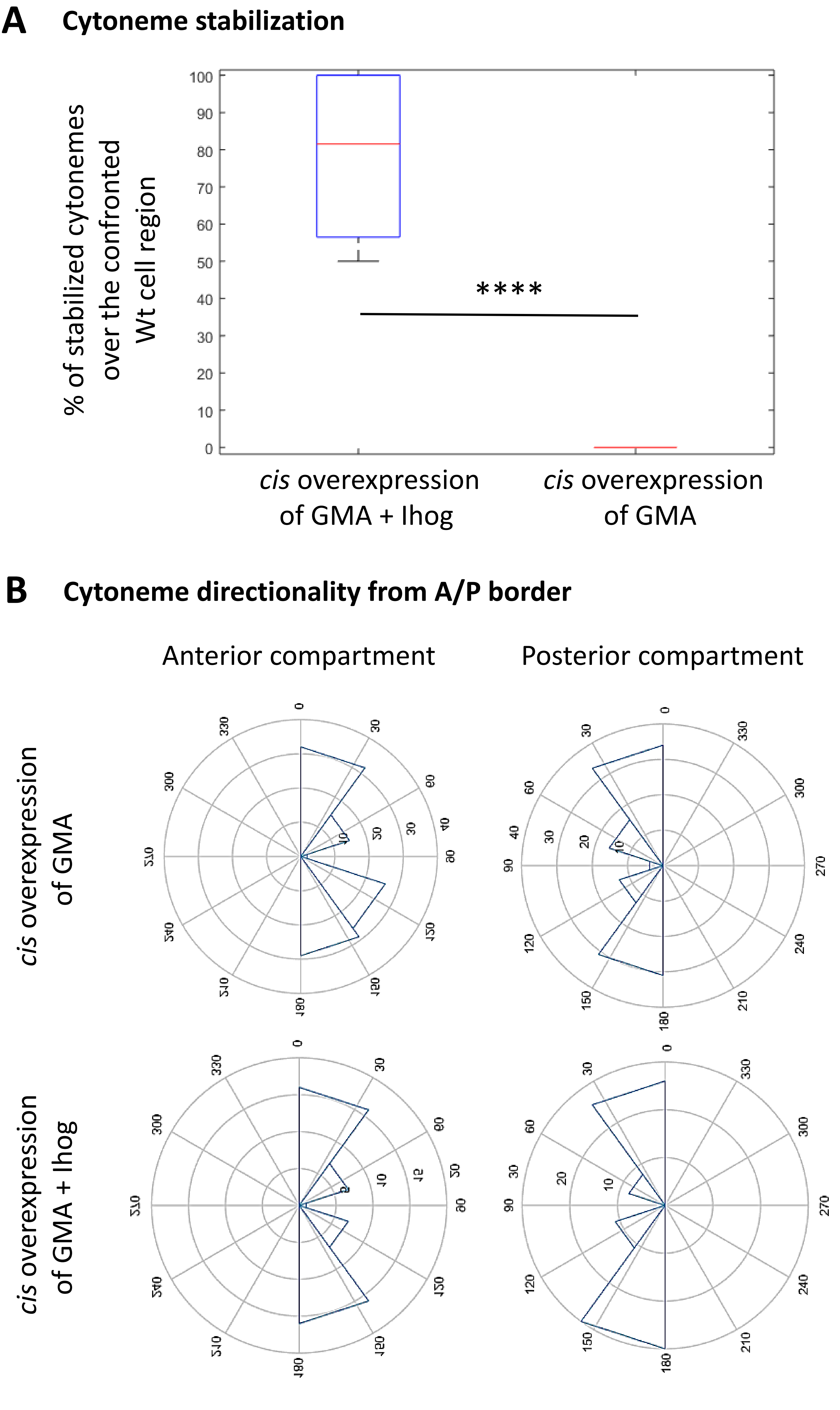
A
Cytoneme stabilization
% of stabilized cytonemes over the confronted Wt cell region
100
90
80
70
60
50
40
30
20
10
0
****
cis overexpression of GMA + Ihog
cis overexpression of GMA
B
Cytoneme directionality from A/P border
Anterior compartment
Posterior compartment
cis overexpression of GMA
cis overexpression of GMA + Ihog

Supplementary Figure 1. Cytoneme stabilization and orientation in abdominal histoblast nets. A) Boxplot and statistical study of the % of P stabilized cytonemes coming from overexpressing Ihog cells and wild type cells, labeled with GMA-GFP a maker generated by a moesin domain tagged with GFP. B) Rose polar diagrams of cytoneme orientation from A and P compartment cell under wild type (up) and Ihog overexpression (down). Statistical analysis, using a Wilcoxon rank sum test per pairs, showed no statistical differences in angle orientation between those conditions in both A and P compartments. The experimental data for the statistical study showed in this figure was obtained from [11]. Raw data (n=4 pupae per genotype, up to 23 cytonemes each pupa) and p-values (ns: $p > 0.05$; *: $p \leq 0.05$; **: $p \leq 0.01$; ***: $p \leq 0.001$; ****: $p \leq 0.0001$) are provided as a Source data file. In box plots, a box indicates the median (in red) and 25 and 75 percentiles, whiskers indicate range of data and crosses indicate outliers.

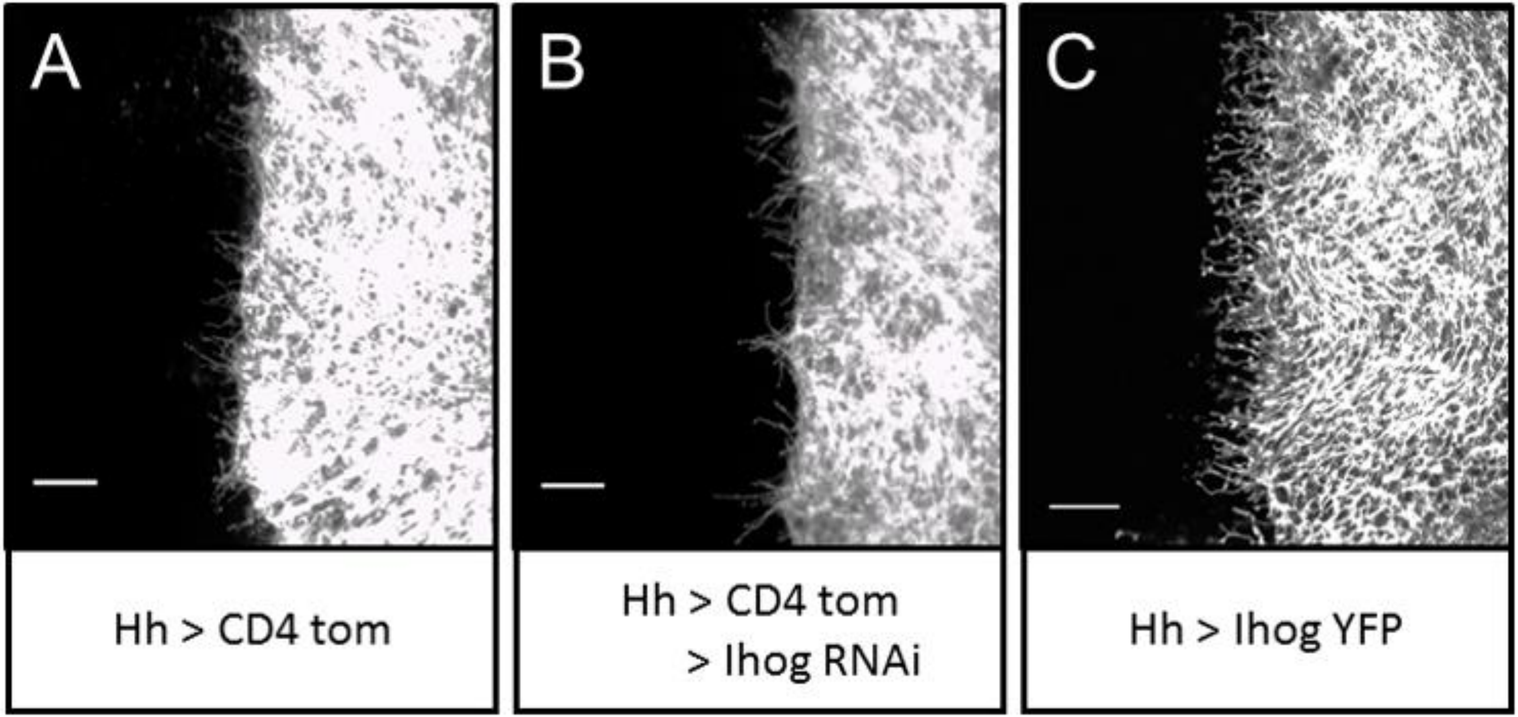
A
B
C
Hh > CD4 tom
Hh > CD4 tom
> Ihog RNAi
Hh > Ihog YFP

Supplementary Figure 2. Orientation of posterior compartment cytonemes under different Ihog levels. A) P compartment cytonemes in wild type Ihog levels marked with CD4-Tomato are oriented to Hh receiving cells. B) Cytonemes in conditions of Ihog *cis*-downregulation in the P compartment, marked with CD4-tomato. C) Cytonemes in conditions of Ihog-YFP *cis*-upregulation in the P compartment cells. Note that Ihog overexpression allows the better visualization of cytonemes, due to their stabilization, but it does not change the orientation towards Hh receiving cells. Representative images in the figure were selected over a sample size of n_{CD4tom}=21, $n_{CD4tom + IhogRNAi}$=23 and $n_{Ihog\ YFP}$ =20. Scale bars: 15μm.

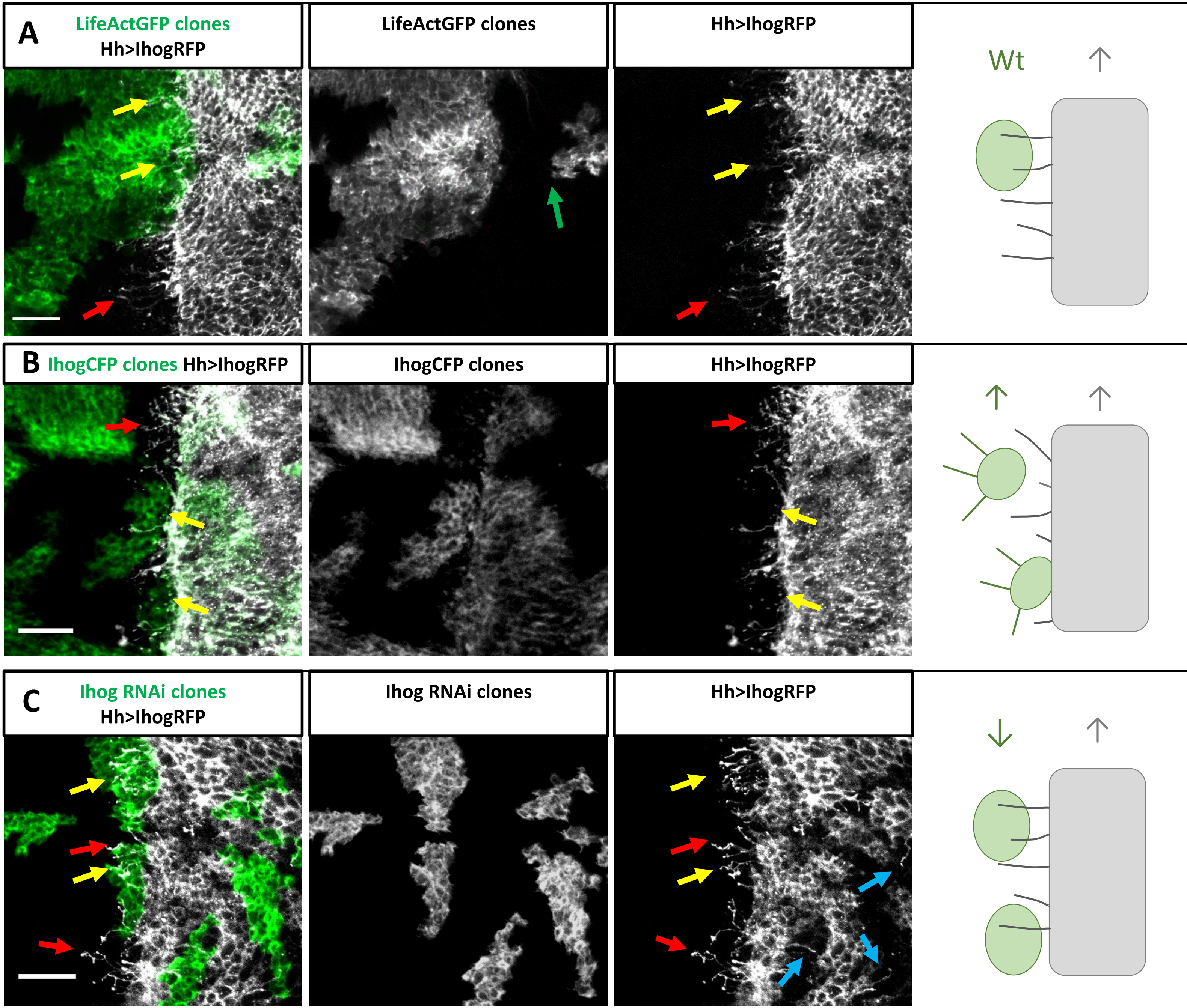
A
LifeActGFP clones
Hh>IhogRFP
LifeActGFP clones
Hh>IhogRFP
Wt
B
IhogCFP clones Hh>IhogRFP
IhogCFP clones
Hh>IhogRFP
C
Ihog RNAi clones
Hh>IhogRFP
Ihog RNAi clones
Hh>IhogRFP

Supplementary Figure 3. Cytoneme stabilization depends on Ihog levels in confronted cell populations. Red arrows point to cytonemes not confronted to clones and yellow arrows point to cytonemes confronted to clones. A) Cytonemes protruding from the P compartment cells overexpressing Ihog confronted to wild type Ihog levels clones (marked clones with LifeActGFP, green) show stabilization. Blue arrow shows the absence of stabilization inside P compartment. B) Cytonemes protruding from the P compartment cells overexpressing Ihog confronted to clones also overexpressing Ihog (green) are not stabilized. C) Cytonemes protruding from the P compartment cells overexpressing Ihog confronted with clones where Ihog is downregulated (green) show stabilization. Blue arrows point to cytoneme stabilization inside P compartment when confronted to clones expressing Ihog RNAi. Representative images in the figure were selected over a sample size of $n_{LifeActGFP}$=17, $n_{Ihog\ clones}$ =23 and $n_{IhogRNAi\ clones}$=21. Scale bars: 15μm.

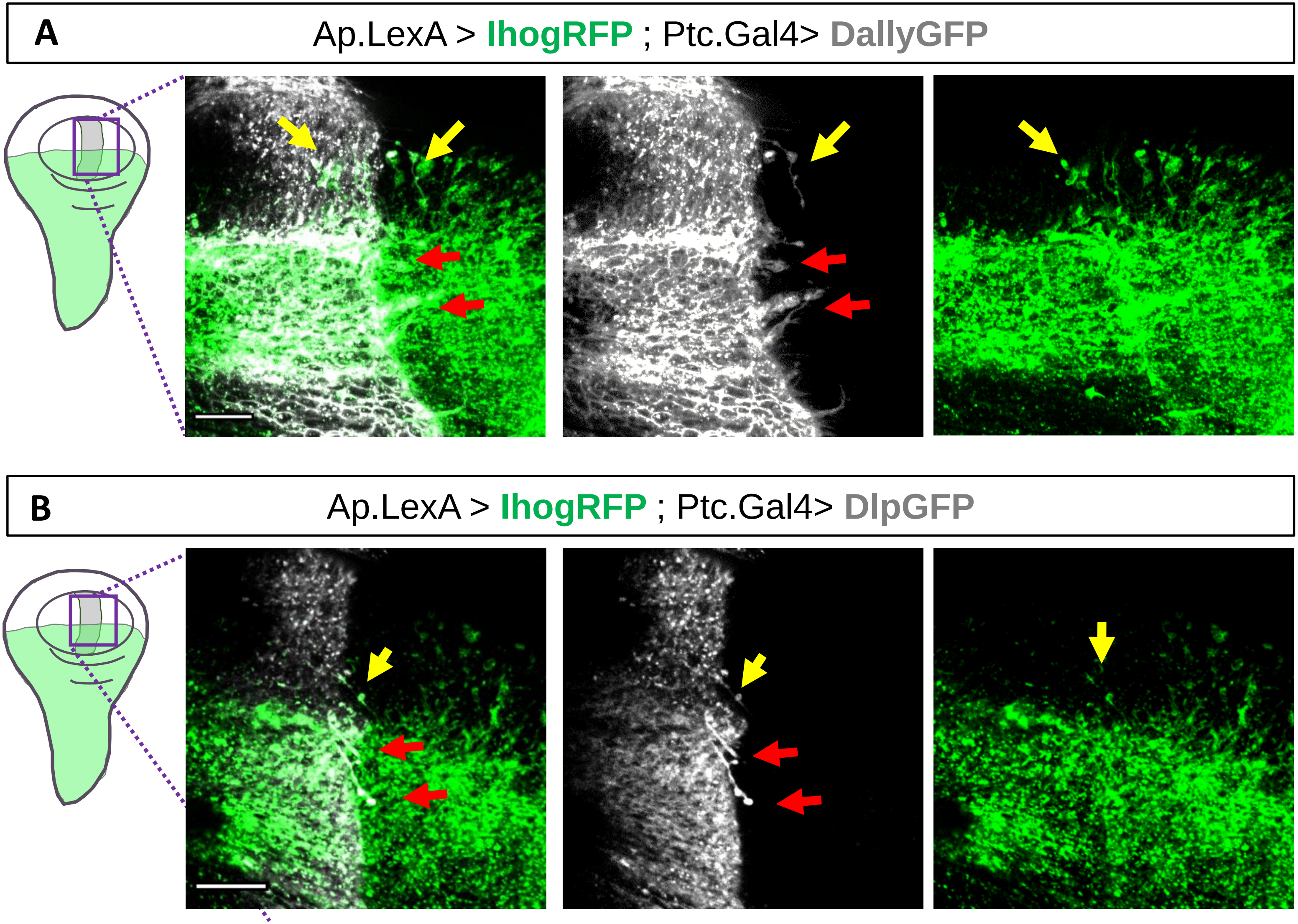
A
Ap.LexA > IhogRFP ; Ptc.Gal4> DallyGFP
B
Ap.LexA > IhogRFP ; Ptc.Gal4> DlpGFP

Supplementary Figure 4. Molecular competition between the available Ihog and glypicans for cytoneme stabilization and orientation. A) Cytoneme stabilization is recovered when an Ihog + Dally co-expressing region is confronted to a region where only Ihog is overexpressed in *trans* (red arrows). Cytonemes changed their orientation to reach regions with confronted levels of Ihog and Dally (yellow arrows). B) Cytoneme stabilization is partially recovered when an Ihog + Dlp co-expressing region is confronted to a region where only Ihog is overexpressed in *trans* (red arrows). Cytonemes changed their orientation to reach regions with confronted levels of Ihog and Dlp (yellow arrows). Representative images in the figure were selected over a sample size of $n_{Ihog+Dally}$=15 and $n_{Ihog+Dlp}$=11. Scale bars: 15µm.

Supplementary Note

Mathematical-physical model for the evolution of continuous cytonemes

In this supplementary material, we derive the mathematical-physical model for the evolution of continuous cytonemes from first principles. Later, we introduce a discretized version of the model that has been useful for the numerical simulations. We end this section with some discussion on the numerical methods along with a description of the parameters of the model that we have set according to experimental evidence.

1. Model derivation

In this part, we shall introduce our mathematical-physical model of cytonemes-based cell communication. We will present our approach in three main parts. We first present the vector field that is responsible for the appropriate cytonemes orientation. Second, we derive the equations of motion of cytonemes from first principles in terms of a constrained variational problem. Finally, we address the propagation of morphogen along those structures.

1.1. Orientation field

Following the discussion provided in the Results Section of the paper, let $\rho(\boldsymbol{r},t)$ be the total "charge" density at position $\boldsymbol{r} \in \mathbb{R}^2$ of the tissue and time t, $\phi(\boldsymbol{r},t)$ the orientation potential generated by the source $\rho(\boldsymbol{r},t)$, and $\boldsymbol{O}(\boldsymbol{r},t)$ the vector field that orients cytonemes. Their expressions are schematized as follows:

$$\begin{aligned}\rho(\boldsymbol{r},t) = \beta_{\text{Ihog}}&\left([\text{Ihog}](\boldsymbol{r}) + \sum\nolimits_{\text{cyt}} [\text{Ihog}]_{\text{cyt}}(\boldsymbol{r},t)\delta_{\text{cyt}}(\boldsymbol{r},t)\right)\\ &+ \beta_{\text{Dlp}}\left([\text{Dlp}](\boldsymbol{r}) + \sum\nolimits_{\text{cyt}} [\text{Dlp}]_{\text{cyt}}(\boldsymbol{r},t)\delta_{\text{cyt}}(\boldsymbol{r},t)\right) \\ &+ \beta_{\text{Dally}}\left([\text{Dally}](\boldsymbol{r}) + \sum\nolimits_{\text{cyt}} [\text{Dally}]_{\text{cyt}}(\boldsymbol{r},t)\delta_{\text{cyt}}(\boldsymbol{r},t)\right),\end{aligned} \quad (1)$$

$$\phi(\boldsymbol{r},t) = (K * \rho)(\boldsymbol{r},t) = \int K(|\boldsymbol{r}-\boldsymbol{s}|)\rho(\boldsymbol{s},t)\, d\boldsymbol{s} \quad (2)$$

$$\boldsymbol{O}(\boldsymbol{r},t) = -\boldsymbol{\nabla}\phi(\boldsymbol{r},t) = -\int \boldsymbol{\nabla}K\,(|\boldsymbol{r}-\boldsymbol{s}|)\rho(\boldsymbol{s},t)\, d\boldsymbol{s}. \quad (3)$$

Let us recall that, as mentioned before, $[X](\boldsymbol{r})$ is static and represents the background concentation of a given protein X (Ihog, Dlp or Dally) at position $\boldsymbol{r} \in \mathbb{R}^2$ associated to basal membranes of tissue cells. On the other hand, $[X]_{\text{cyt}}(\boldsymbol{r},t)\delta_{\text{cyt}}(\boldsymbol{r},t)$ is dynamic and describes the linear density of such a protein along cytonemes, and $\sum_{\text{cyt}}$ refers to the sum over all cytonemes. We observe that, despite $\rho(\boldsymbol{r},t)$ having mass density units, it has not positive sign throughout the full extracellular matrix due to the choice of signs of coefficients $\beta_{\text{Ihog}} > 0$ and

$\beta_{\mathrm{Dlp}}, \beta_{\mathrm{Dally}} < 0$. As we will illustrate later, this quantity will play a similar role like a "charge" density in the physical theory of electric potentials.

Using $\rho(\boldsymbol{r}, t)$ as source, we construct the associated orientation potential $\phi(\boldsymbol{r}, t)$ given by superposition of a static volume potential coming from the background source levels over basal membranes, and multiple single layer potentials along each of the non-static cytonemes in the system. At this point, let us emphasize again that, according to the experimental evidences discussed in the main text, cytonemes strongly interact within a distance of around 5 μ. Hence, the interaction potential is modeled in terms of a kernel function that is compactly supported in such region:

$$K'(r) = \begin{cases} \frac{h\delta_0^\alpha}{r^\alpha}, & r \in (0, \delta_0), \\ h, & r \in [\delta_0, \delta_1), \\ \frac{h}{1+\exp\left(\frac{2r-(\delta_1+\delta_2)}{(\delta_2-r)(r-\delta_1)}\right)}, & r \in [\delta_1, \delta_2), \\ 0, & r \in [\delta_2, \infty), \end{cases} \tag{4}$$

where $r = |\boldsymbol{r}|$. See also the plot below for the values $h = 0.01\ \mu^2\ \mathrm{st}^{-2}\ \mathrm{mass}^{-1}$, $\alpha = 0.5$, $\delta_0 = 0.5\ \mu$, $\delta_1 = 4\ \mu$ and $\delta_2 = 5\ \mu$, and Section 4 for a complete table of parameters used in the simulations. Note also that the experiments do not show any apparent correlation between cytonemes acceleration, and the morphogen gradient orientation. Hence, we have assumed a constant interaction intensity h in the orientation potential inside the interaction region $r \in [\delta_0, \delta_1)$. We have also included the distances δ_0 and δ_2, in order to capture attraction/repulsion and smooth transitions to dead zones without interactions, respectively.

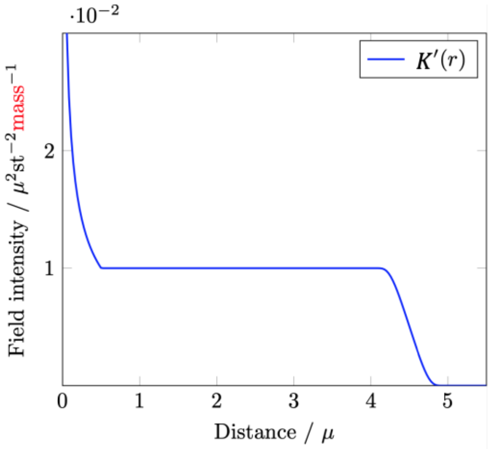

Note that the orientation field $\boldsymbol{O}(\boldsymbol{r}, t)$ takes the form of a classical electric field associated to a charge distribution $\rho(\boldsymbol{r}, t)$. It represents the force felt by a punctual test charge with unitary modulus and positive sign. Then, the force exerted on a particle with charge Q at position $\boldsymbol{r} \in \mathbb{R}^2$ and time t is given by $|Q\ \boldsymbol{O}(\boldsymbol{r}, t)|$ and the direction and orientation will come out from the sign of the product between Q and the charge density $\rho(\boldsymbol{r}, t)$, containing the weighted information of the concentration of the three different proteins.

In order to understand the final attractive/repulsive character of interactions, we shall look at the profile of the concentration levels of each of the involved protein (Ihog, Dlp and Dally) throughout the ECM. To simplify the representation, we introduce a simpler smoothed diagram for the average of concentration levels at different sections of tissue parallel to the dorsoventral axis (see Fig. 5 in the main document for an experimental plot of these concentrations)

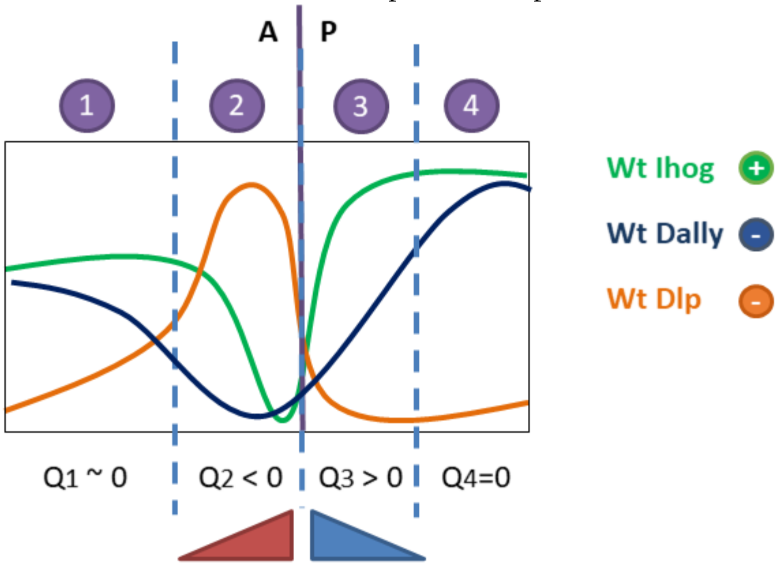

In concordance with the experimental evidence discussed in the main text, the concentrations are present in four interaction regions:

- **Region 1)** Anterior region far away from the A/P boundary: the interactions are negligible due to both high levels of Ihog and Dally (i.e., the total "charge" in the region is $Q_1 \approx 0$).
- **Region 2)** Anterior region close to the A/P boundary: the cytonemes are mostly charged by Dlp and Dally (total "charge" negative, $Q_2 < 0$).
- **Region 3)** Posterior region close to the A/P boundary: the cytonemes are mostly charged by Ihog (total "charge" $Q_3 > 0$).
- **Region 4)** Posterior region far away from the A/P boundary: the interactions are negligible due to both high levels of Ihog and Dally (i.e., the total "charge" in the region is $Q_4 \approx 0$).

The difference in the concentration levels between regions 2 and 3 (i.e., the intense orientation field generated by the difference in charges at the boundary) forms stable interactions of cytonemes coming from both the anterior and posterior compartments.

1.2. Cytonemes kinetics

As discussed before, the kinetics of cytonemes is a complex mechanism since we need to account for many geometric constraints of the system. As a side-effect, morphogen proteins cannot propagate freely across the ECM following the orientation field, but they are subject to the 1D nature of filipodia. In order to model it, we shall define mathematically a cytoneme as a parametrized curve $\boldsymbol{\gamma}(\xi, t)$ with "charge" distribution $\bar{\rho}(\boldsymbol{r}, t)$ subject to the constraint

$$|\boldsymbol{\gamma}'(\xi, t)| = 1\,. \quad (5)$$

Since cytonemes can elongate and retract by (de)polymerization of actin dimers, the length $L(t)$ of the curve $\boldsymbol{\gamma}(\xi,t)$ also evolves in time. In particular, the length is given by

$$\int_0^{L(t)} |\boldsymbol{\gamma}'(\xi,t)| d\xi = L(t) = L(0) + vt\,. \quad (6)$$

Here, we have taken into account the constraint (5) and v is the elongation/retraction speed of cytonemes. Define the Lagrangian associated to the kinetic and potential energy for the dynamics of the cytoneme

$$\mathcal{L}(\xi,t,\boldsymbol{\gamma},\boldsymbol{\gamma}',\dot{\boldsymbol{\gamma}}) = \underbrace{\frac{1}{2}|\dot{\boldsymbol{\gamma}}|^2}_{\text{kinetic energy}} - \underbrace{\bar{\rho}\phi(\boldsymbol{\gamma},t)}_{\text{potential energy}}\,. \quad (7)$$

The equations of motion for such a cytoneme can then be obtained by minimizing the constrained action functional

$$\min_{\boldsymbol{\gamma}\in\mathcal{D}} \mathcal{A}[\boldsymbol{\gamma}] = \min_{\boldsymbol{\gamma}\in\mathcal{D}} \int_0^T \int_0^{L(t)} \mathcal{L}\big(\xi,t,\boldsymbol{\gamma}(\xi,t),\boldsymbol{\gamma}'(\xi,t),\dot{\boldsymbol{\gamma}}(\xi,t)\big)\, d\xi\, dt\,, \quad (8)$$
$$\mathcal{D} = \left\{\gamma \in C^1 : \left|\boldsymbol{\gamma}'(\xi,t)\right| = 1, + \text{boundary conditions}\right\}.$$

Its solution is governed by the Euler-Lagrange equations for the modified Lagrangian $\mathcal{L}^* = \mathcal{L} + \frac{1}{2}\lambda(\xi,t)|\boldsymbol{\gamma}'|^2$, i.e.,

$$\frac{\partial}{\partial t}\frac{\partial\mathcal{L}^*}{\partial\dot{\boldsymbol{\gamma}}} + \frac{\partial}{\partial\xi}\frac{\partial\mathcal{L}^*}{\partial\boldsymbol{\gamma}'} - \frac{\partial\mathcal{L}^*}{\partial\boldsymbol{\gamma}} = 0, \quad (9)$$

with $\lambda(\xi,t)$ the Lagrange multiplier associated to the constraint (5). The boundary conditions are given by

$$\begin{cases} \boldsymbol{\gamma}(0,t) = \boldsymbol{r_0}, & t \in [0,T], \\ \boldsymbol{\gamma}(\xi,0) = \boldsymbol{\gamma_0}(\xi), & \xi \in [0,L(0)], \\ \dot{\boldsymbol{\gamma}}(\xi,0) = \dot{\boldsymbol{\gamma}}_{\boldsymbol{0}}(\xi), & \xi \in [0,L(0)]. \end{cases} \quad (10)$$

Here, $\boldsymbol{r_0}$ is the basis where the cytoneme is attached to the cell membrane and $\boldsymbol{\gamma_0}(\xi)$ and $\dot{\boldsymbol{\gamma}}_{\boldsymbol{0}}(\xi)$ are its initial shape and velocity configurations. In (10) there is still a boundary condition missing at the tip of the cytoneme (i.e., when $\xi = L(t)$ for all $t \in [0,T]$), see red line in next figure:

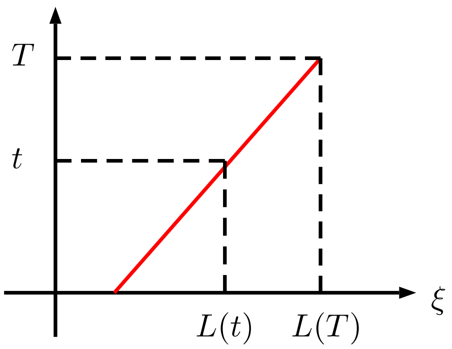

A natural closure is obtained by choosing *free boundary* conditions in the resolution of the variational problem (8), that is,

$$\frac{\partial \mathcal{L}^*(\xi, t, \boldsymbol{\gamma}, \boldsymbol{\gamma}', \dot{\boldsymbol{\gamma}})}{\partial(\boldsymbol{\gamma}', \dot{\boldsymbol{\gamma}})}\Big|_{\xi=L(t)} \cdot \left(1, -\dot{L}(t)\right) = 0. \quad (11)$$

To summarize, cytoneme $\boldsymbol{\gamma}(\xi, t)$ evolves according to the system

$$\begin{cases} \dfrac{\partial}{\partial t}(\dot{\boldsymbol{\gamma}}) = \dfrac{\partial}{\partial \xi}(\lambda \boldsymbol{\gamma}') - \dfrac{1}{\tau}\dot{\boldsymbol{\gamma}} - \bar{\rho}\boldsymbol{\nabla}\phi(\boldsymbol{\gamma}, t), \\ \boldsymbol{\gamma}(0,t) = \boldsymbol{r_0}, \\ \boldsymbol{\gamma}(\xi, 0) = \boldsymbol{\gamma_0}(\xi) \text{ and } \dot{\boldsymbol{\gamma}}(\xi, 0) = \dot{\boldsymbol{\gamma}}_{\boldsymbol{0}}(\xi), \\ |\boldsymbol{\gamma}'(\xi, t)| = 1, \\ v\dot{\boldsymbol{\gamma}}(L(t), t) + \lambda(L(t), t)\, \boldsymbol{\gamma}'(L(t), t) = 0. \end{cases} \quad (12)$$

In (12) we have introduced the effect of friction with the extracellular matrix through a linear term $\frac{1}{\tau}\dot{\boldsymbol{\gamma}}$, thus reducing fluctuations.

The resulting system is a forced damped nonlinear wave equation for the evolution of each cytoneme, where tension can be identified with the Lagrange multiplier $\lambda(\xi, t)$. However, this system is difficult to analyze because the Lagrange multipler is involved in it and it is not clear how to deal with both unknown quantities $\lambda(\xi, t)$ and $\boldsymbol{\gamma}(\xi, t)$. Then, to manage this problem, a useful idea is to find an appropriate system of generalized coordinates that remove the presence of multipliers.

2. Model discretization

After introducing the change of variables

$$\boldsymbol{\gamma}'(\xi, t) = (\cos\theta(\xi, t), \sin\theta(\xi, t)), \quad (13)$$

that restates the constraint (5), we can eliminate the Lagrange multiplier $\lambda(\xi,t)$ by integrating the first equation in (12) from $\xi = 0$ to $\xi = L(t)$, using the last free boundary condition in the integration by parts and finally multiplying by $\boldsymbol{\gamma'}^{\perp}$, obtaining

$$
\begin{aligned}
\frac{\partial}{\partial t}\int_{\xi}^{L(t)}\int_{0}^{\xi'} \cos\big(\theta(\xi'',t)-\theta(\xi,t)\big)\dot{\theta}(\xi'',t)\, d\xi''\, d\xi' \\
-\int_{\xi}^{L(t)}\int_{0}^{\xi'} \sin\big(\theta(\xi'',t)-\theta(\xi,t)\big)\dot{\theta}(\xi'',t)\dot{\theta}(\xi,t)d\xi'' d\xi' \\
= -\frac{1}{\tau}\int_{\xi}^{L(t)}\int_{0}^{\xi'} \cos\big(\theta(\xi'',t)-\theta(\xi,t)\big)\dot{\theta}(\xi'',t)d\xi'' d\xi' \\
+\int_{\xi}^{L(t)} \sin\theta(\xi,t)(\partial_x\phi)(\boldsymbol{\gamma}(\xi',t),t)\,\bar{\rho}\, d\xi' \\
-\int_{\xi}^{L(t)} \cos\theta(\xi,t)\big(\partial_y\phi\big)(\boldsymbol{\gamma}(\xi',t),t)\,\bar{\rho}\, d\xi' \ . \quad (14)
\end{aligned}
$$

For numerical purposes, we shall discretize the above continuous description. In this way, the continuous mathematical curve $\boldsymbol{\gamma}(\xi,t)$ becomes a chain of particles at positions $\boldsymbol{r_i}(t) \approx \boldsymbol{\gamma}(\xi_i(t),t)$, where

$$
\begin{cases}
\boldsymbol{r_i}(t) = \boldsymbol{r_0} + \sum_{j=1}^{j=i} l_j(t)\left(\cos\theta_j(t), \sin\theta_j(t)\right), & i = 1 \ldots, N(t), \\
\xi_i(t) = \sum_{j=1}^{j=i} l_j(t), & i = 1, \ldots, N(t).
\end{cases} \quad (15)
$$

Here, the number of particles in the chain, $N(t)$, is time-dependent due to the inherent elongation/retraction dynamics described in the free boundary condition in (12). Specifically, we shall describe it in terms of a jump function at some discrete values of time

$$t_k := \frac{l}{|v|}k, \ \ k \in \mathbb{Z}_0^+. \quad (16)$$

Specifically, $N(t)$ and lengths $l_i(t)$ of bonds in the chain are given by the following expressions according to the case $v > 0$ (elongation) or $v < 0$ (retraction):

DISCRETE JUMPS: $t_{k-1} < t < t_k$,	
**ELONGATION DYNAMICS** ($v > 0$)	**RETRACTION DYNAMICS** ($v < 0$)
$N(t) = N_0 + k$	$N(t) = N_0 - (k-1)$
$l_i(t) = \begin{cases} l, & i = 1, \dots, N_0 + k - 1 \\ tv - (k-1)l, & i = N_0 + k \end{cases}$	$l_i(t) = \begin{cases} l, & i = 1, \dots, N_0 - k \\ kl + tv, & i = N_0 - (k-1) \end{cases}$

That is, all the bonds have fixed length l except for the last one, containing the tip, that grows (or shrinks) at constant velocity until it achieves the maximal (minimal) length and a new jump takes place.

This gives rise to the discrete version of (14)

$$\boldsymbol{M}(\boldsymbol{\Theta}(t), t)\ddot{\boldsymbol{\Theta}}(t)^T = -\frac{1}{\tau}\boldsymbol{M}(\boldsymbol{\Theta}(t), t)\dot{\boldsymbol{\Theta}}(t)^T + \boldsymbol{G}\big(\boldsymbol{\Theta}(t), \dot{\boldsymbol{\Theta}}(t), t\big)^T, \qquad t_{k-1} < t < t_k . \quad (17)$$

Here, we denote $\boldsymbol{\Theta}(t) = \left(\theta_1(t), \dots, \theta_{N(t)}(t)\right) \in \mathbb{R}^{N(t)}$, $\dot{\boldsymbol{\Theta}}(t) = \left(\dot{\theta}_1(t), \dots, \dot{\theta}_{N(t)}(t)\right) \in \mathbb{R}^{N(t)}$ and the matrix $\boldsymbol{M}(\boldsymbol{\Theta}(t), t) = \left(M_{ij}(\boldsymbol{\Theta}(t))\right) \in \mathcal{M}_{N(t)\times N(t)}(\mathbb{R})$ and vector $\boldsymbol{G}\big(\boldsymbol{\Theta}(t), \dot{\boldsymbol{\Theta}}(t), t\big) = \left(G_i\big(\boldsymbol{\Theta}(t), \dot{\boldsymbol{\Theta}}(t), t\big)\right) \in \mathbb{R}^{N(t)}$ read

$$M_{ij}(\boldsymbol{\Theta}(t), t) = \bar{\rho}\left(\sum_{k=\max\{i,j\}}^{N(t)} l_k(t) l_j(t)\right)\cos(\theta_i(t) - \theta_j(t)), \quad (18)$$

$$G_i\big(\boldsymbol{\Theta}(t), \dot{\boldsymbol{\Theta}}(t), t\big) = -v\bar{\rho}\sum_{j=1}^{N(t)} l_j(t)\dot{\theta}_j(t)\cos\left(\theta_j(t) - \theta_i(t)\right) + \sum_{j=i}^{N(t)} l_j(t)\big(\sin\theta_i(t)(\partial_x\phi)\big(r_j(t), t\big) - \cos\theta_i(t)(\partial_y\phi)\big(r_j(t), t\big) + \bar{\rho}\sum_{k=1}^{j} l_k(t)\dot{\theta}_k^2(t)\sin(\theta_k(t) - \theta_i(t))\big). \quad (19)$$

Note that in the discretization we are assuming that the charge distribution $\bar{\rho}$ is constant along the cytoneme, that is, equally distributed in concentrations of Ihog, Dally and Dlp, for all time t.

3. Numerics

The system (17) has been solved by firstly inverting the matrix $M(\boldsymbol{\Theta}(t), t)$ with LU-decomposition. The time evolution has been solved with a fourth order Runge-Kutta method, for each cytoneme of the numerical simulations. The concentrations involved in the computation of the charge density ρ have been obtained from the pixel intensities in each channel of the experiments, in gray-scale units.

4. Parameters

The parameters have been calculated by direct evaluation in the experiments:

PARAMETER	VALUE
β_{Ihog}	1 a.u.
β_{Dlp}	-1 a.u.
β_{Dally}	-1 a.u.
α	0.5 a.u.
K	$0.01\ \mu^2\ \mathrm{st}^{-2}\ \mathrm{mass}^{-1}$
δ_0	$0.5\ \mu$
δ_1	$4\ \mu$
δ_2	$5\ \mu$
l	$0.05\ \mu$
v	$1\ \mu\ \mathrm{st}^{-1}$
τ	0.01 st

where st stands for "simulation timescale".
