# Supplementary material for: Predictive model for cytoneme guidance in Hedgehog signaling based on Ihog- Glypicans interaction
Source: Nat Commun. 2022 Sep 26;13:5647. doi: 10.1038/s41467-022-33262-4 (PMC9512826; doi:10.1038/s41467-022-33262-4)
Supplement: Supplementary file 3 — Description of Additional Supplementary Files [file 41467_2022_33262_MOESM3_ESM.pdf]

Description of Additional Supplementary Files

File Name: Supplementary Movie 1
Description: **Cytoneme stabilization in wing imaginal disc due to Ihog overexpression.** The movie shows cytoneme temporal stabilization in ex vivo wing imaginal disc. Most of cytoneme population was "frozen" with a small fraction of dynamic cytonemes (cytoneme 1, blue dot). This result is consistent with previous studies in abdominal histoblast nests and corroborates by comparison the Ihog stabilization effect in different Drosophila tissues.

File Name: Supplementary Movie 2
Description: **Lack of cytoneme stabilization after confronting two cell populations overexpressing Ihog.** The movie shows dynamic cytonemes coming from A (green) and P (grey) cells, both compartments overexpressing Ihog. The average lifetime of the marked cytonemes was 9-12 min, similar to the wild type dynamics [11].

File Name: Supplementary Movie 3
Description: **Ihog pattern from apical to basal side of the wing imaginal disc epithelium**. The movie shows the Ihog protein distribution in green (Bac.Ihog.GFP) and the pathway receptor Ptc expression pattern in red (EnhancerPtcRed). Note that Ihog downregulation correlates with the Hh receiving area (marked by Ptc expression). Furthermore, this downregulation is stronger in the basal side of the wing imaginal disc, where the cytonemes are present.

File Name: Supplementary Movie 4
Description: ***In silico* cytoneme guidance behavior in confronted cell population overexpressing Ihog.** Simulation of the behavior of cytonemes protruding from the P compartment cells overexpressing Ihog (red) and confronted to an Ihog overexpressing clone (grey).

File Name: Supplementary Movie 5
Description: ***In silico* cytoneme guidance behavior when confronted to ectopic Dally clones.** Simulation of the behavior of cytonemes protruding from the P compartment cells overexpressing Ihog (red) and confronted to Dally overexpressing clone (green).
File Name: Supplementary Movie 6
Description: ***In silico* cytoneme guidance in wild type Hh receiving zone.** Simulation of cytonemes emanating from the A (red) and P (green) compartment cells in the wild type wing disc that has normal distribution of Ihog, Dally and Dlp proteins.

File Name: Supplementary Movie 7
Description: **Numerical resolution of cytoneme orientation.** The movie shows an example of growing cytonemes modeled by numerical nodes along the cytoneme length. All nodes sense the field spatial and temporally in each software iterations. This last property is shown in the video by the re-colocalization of nodes after manually changing one cytoneme trajectory.
