# Supplementary material for: Predictive model for cytoneme guidance in Hedgehog signaling based on Ihog- Glypicans interaction
Source: Nat Commun. 2022 Sep 26;13:5647. doi: 10.1038/s41467-022-33262-4 (PMC9512826; doi:10.1038/s41467-022-33262-4)
Supplement: Supplementary file 11 — Reporting Summary [file 41467_2022_33262_MOESM11_ESM.pdf]

nature portfolio

Corresponding author(s): Isabel Guerrero, Juan Soler

Last updated by author(s): Isabel Guerrero

Reporting Summary

Nature Portfolio wishes to improve the reproducibility of the work that we publish. This form provides structure for consistency and transparency in reporting. For further information on Nature Portfolio policies, see our Editorial Policies and the Editorial Policy Checklist.

Statistics

For all statistical analyses, confirm that the following items are present in the figure legend, table legend, main text, or Methods section.

n/a	Confirmed	
☐	☒	The exact sample size (*n*) for each experimental group/condition, given as a discrete number and unit of measurement
☐	☒	A statement on whether measurements were taken from distinct samples or whether the same sample was measured repeatedly
☐	☒	The statistical test(s) used AND whether they are one- or two-sided<br>*Only common tests should be described solely by name; describe more complex techniques in the Methods section.*
☐	☒	A description of all covariates tested
☐	☒	A description of any assumptions or corrections, such as tests of normality and adjustment for multiple comparisons
☐	☒	A full description of the statistical parameters including central tendency (e.g. means) or other basic estimates (e.g. regression coefficient) AND variation (e.g. standard deviation) or associated estimates of uncertainty (e.g. confidence intervals)
☐	☒	For null hypothesis testing, the test statistic (e.g. *F*, *t*, *r*) with confidence intervals, effect sizes, degrees of freedom and *P* value noted<br>*Give P values as exact values whenever suitable.*
☒	☐	For Bayesian analysis, information on the choice of priors and Markov chain Monte Carlo settings
☒	☐	For hierarchical and complex designs, identification of the appropriate level for tests and full reporting of outcomes
☒	☐	Estimates of effect sizes (e.g. Cohen's *d*, Pearson's *r*), indicating how they were calculated

Our web collection on statistics for biologists contains articles on many of the points above.

Software and code

Policy information about availability of computer code

Data collection	Software source code is available in: https://github.com/AdrianA-T/Guidance-Model and was written in C# using Unity Engine
Data analysis	Data were quantified using Fiji 2.6.0 and analysis were performed using Matlab2015a, further details are in Materials and Methods and upon request.

For manuscripts utilizing custom algorithms or software that are central to the research but not yet described in published literature, software must be made available to editors and reviewers. We strongly encourage code deposition in a community repository (e.g. GitHub). See the Nature Portfolio guidelines for submitting code & software for further information.

Data

Policy information about availability of data

All manuscripts must include a data availability statement. This statement should provide the following information, where applicable:

- Accession codes, unique identifiers, or web links for publicly available datasets
- A description of any restrictions on data availability
- For clinical datasets or third party data, please ensure that the statement adheres to our policy

Source data generated in this study is provided with this paper in Source Data file and more supplementary information from the corresponding authors upon request.

Human research participants

Policy information about studies involving human research participants and Sex and Gender in Research.

Reporting on sex and gender	N/A since not human research was perfomed.
Population characteristics	N/A since not human research was perfomed.
Recruitment	N/A since not human research was perfomed.
Ethics oversight	N/A since not human research was perfomed.

Note that full information on the approval of the study protocol must also be provided in the manuscript.

Field-specific reporting

Please select the one below that is the best fit for your research. If you are not sure, read the appropriate sections before making your selection.

☒ Life sciences ☐ Behavioural & social sciences ☐ Ecological, evolutionary & environmental sciences

For a reference copy of the document with all sections, see nature.com/documents/nr-reporting-summary-flat.pdf

Life sciences study design

All studies must disclose on these points even when the disclosure is negative.

Sample size	The sample size were calculated for each study according to the regular sample size determined in other Drosophila studies (see the references) and taking into account the confidece range for Wilcoxon rank sum test. The exact sample size per study is also detailed in the figure legends of the manuscript, the material and methods and in the source data file.
Data exclusions	No data were excluded.
Replication	All experiments were reproduced to reliably support the conclusions stated in the manuscript. The exact sample size per study is included and detailed in the figure legends of the manuscript, the material and methods and in the source data file.
Randomization	N/A. The fly samples were selected evenly according to the genotype of study.
Blinding	Administration of samples was carried out using coded labels that did not contain the genotype nomenclature. Hence, the acquisition of the image samples in the microscope were a blinded experiment.

Reporting for specific materials, systems and methods

We require information from authors about some types of materials, experimental systems and methods used in many studies. Here, indicate whether each material, system or method listed is relevant to your study. If you are not sure if a list item applies to your research, read the appropriate section before selecting a response.

Materials & experimental systems

n/a	Involved in the study	
☐	☒	Antibodies
☒	☐	Eukaryotic cell lines
☒	☐	Palaeontology and archaeology
☐	☒	Animals and other organisms
☒	☐	Clinical data
☒	☐	Dual use research of concern

Methods

n/a	Involved in the study	
☒	☐	ChIP-seq
☒	☐	Flow cytometry
☒	☐	MRI-based neuroimaging

Antibodies

Antibodies used	The mouse monoclonal α-Dlp primary antibody from (Lum L, Yao S, Mozer B, et al. Identification of Hedgehog pathway components by RNAi in Drosophila cultured cells. Science (80-). 2003;299(5615):2039-2045. doi:10.1126/science.1081403) at 1:30 dilution. The rat monoclonal α-Ci primary antibody from (Motzny CK, Holmgren R. The Drosophila cubitus interruptus protein and its role in the wingless and hedgehog signal transduction pathways. Mech Dev. 1995;52(1):137-150. doi:10.1016/0925-4773(95)00397-J) at

	1:20 dilution.<br>α-GFP (rabbit anti GFP polyclonal antibody from Chromotek, ref : PABG1) at 1:1000 dilution<br>anti-mouse Pacific Blue from ThermoFischer (ref: P-31582) at 1:400 dilution<br>anti-rabbit Alexa 647 from ThermoFischer (ref: A-31573) at 1:400 dilution
Validation	The valitation of the antibodies were performed by: Primary: α-Dlp: doi:10.1126/science.1081403; α-GFP: Chromotek ; α-Ci: doi:10.1016/0925-4773(95)00397-J, Secondary: anti-mouse Pacific Blue: ThermoFischer; anti-rabbit Alexa 647: ThermoFischer. Since our studied proteins have a specific pattern in the tissue, we use that unique distribution to validate that the antibody was working propertly in our samples.

Animals and other research organisms

Policy information about studies involving animals; ARRIVE guidelines recommended for reporting animal research, and Sex and Gender in Research

Laboratory animals	Drosophila melanogaster strains (mutants and transgenes) are described in the manuscript. The fly sample were collected at third instar larvae (L3) witch correspond to three-five days from the egg fertilization.
Wild animals	No wild animals were used in the study.
Reporting on sex	Not applicable
Field-collected samples	No field collected samples were used in the study.
Ethics oversight	The study did not require an ethical approval.

Note that full information on the approval of the study protocol must also be provided in the manuscript.
